# THE BENEFITS OF INTERGENERATIONAL REMINISCENCE AND DST ON THE WELL-BEING OF YOUNG ADULT PARTICIPANTS

**DOI:** 10.1093/geroni/igae098.2735

**Published:** 2024-12-31

**Authors:** Ling Xu, Noelle Fields, Kathryn Daniel, Daisha Cipher, Brooke Troutman

**Affiliations:** The University of Texas at Arlington, Arlington, Texas, United States; The University of Texas at Arlington, Arlington, Texas, United States; The University of Texas Arlington, Arlington, Texas, United States; The University of Texas Arlington, Arlington, Texas, United States; Air Force Academy, Air Force Academy, Colorado, United States

## Abstract

**Objective:**

Intergenerational contact between older people and young adults have many benefits for both young and older generations. An intergenerational reminiscence and DST project was conducted with young adult college students calling older partners each week for 10 weeks. This study reported the benefits of this project in improving the well-being of young adult participants.

**Method:**

A randomized control trial was used to assess the effects of the intervention. Participants of young and older adults were randomly paired and assigned to Reminiscence (n = 55) or sham (Social Wellness, n = 48) group. Data were collected at baseline, mid- intervention, at the end of the intervention. Linear mixed models (LMM) were performed to compare the Social Wellness group with the Reminiscence group on levels of loneliness, attitudes toward aging, and knowledge of Alzheimer’s’ Disease (AD) among the student participants.

**Results:**

Results from LMM revealed no significant effects for group or time on attitudes toward aging. However, the groups significantly differed on their changes over time in loneliness. LMM revealed a significant interaction between group and time, t(101) = -3.06, p =.003. The two groups also significantly differed on their changes over time in knowledge. LMM revealed a significant interaction between group and time, t(101) = 2.51, p =.014.

**Discussion:**

Results suggest that weekly intergenerational engagements with older adults benefit young adult college students with loneliness and knowledge of AD. Implications for research and programs in improving the well-being of young adults are discussed.

